# Hospital and Institutional News

**Published:** 1918-01-19

**Authors:** 


### HOSPITAL AND INSTITUTIONAL NEWS.

### THE NEW DIRECTOR-GENERAL ARMY MEDICAL SERVICES.

SIR ALFRED KEOGH will relinquish the Director-Generalship on March 1 next, having brought into existence a machinery which works admirably and runs smoothly. He will be succeeded by Colonel T. H. J. C. Goodwin, C.M.G., D.S.O., who gave such brilliant proof of high powers of organisation on the Western Front by his administration of the hospital of which he had charge, that under him it became a model of orderliness, discipline, and efficiency. This notable success won the attention of Sir Alfred Keogh, whose capacity for getting the right men to do a particular work is notorious, and gained for Colonel Goodwin the high distinction of being selected for the important appointment of Assistant-Director of Medical Services to the British Recruiting Mission in the United States of America. It was a post to test the qualities of any man, whatever his experience and capacity, and Colonel Goodwin's complete success is demonstrated by a senator there with whom he had much to do, who has publicly stated that Colonel Goodwin proved himself to be "the most tactful, kind-hearted, willing and helpful man" the senator had ever met. "He will talk and discuss things with us for hours, to tell us anything he knows. He is easy of access, not only to medical people, but to all sorts and conditions of men. I should say he must be just worshipped by the men under him." This is the fact, as all who have had the privilege to be associated with the new head of the Army Medical Services cordially agree. Possessed of a quick, dominant personality, he has the inestimable power of charming and winning the confidence of all associated with him. He takes infinite pains to elucidate the facts, is a just and successful disciplinarian, goes to the bottom of things, knows what he wants and gets it. Colonel Goodwin never shirks responsibility, and is an indomitable worker. We are glad to know that he is imbued with the feeling, which we share, that nothing is too good for the British sailor or soldier. The best and nothing less is good enough for our warriors. He has the capacity of taking infinite pains, and will devote time to the weighing of every suggestion, the acceptance or rejection of which rests upon demonstrated merit alone. Considering all things the Nation, the Empire and the Army have good cause to rejoice that Colonel Goodwin's hard work and genuine success have won for him the high post of Director-General of the Army Medical Services, in which office the hopes of many will follow him with an assured confidence of his complete success.

### SIR ROBERT ARUNDELL HUDSON, G.B.E.

SIR ROBERT ARUNDELL HUDSON, who was made a Grand Cross of the Order of the British Empire in the New Year Honours' List, is the Chairman of the Finance Committee of the British Red Cross Society and Order of St. John. At the outbreak of the war he was Chief Agent to the Liberal Party and his services were loaned to the Red Cross for the duration of the war. As Chairman of the Finance Committee he has a two-fold responsibility of great importance. First, in a large degree he is responsible for the raising of the "Times" Fund, and secondly for the direction of the expenditure of vast sums in every theatre of war throughout the world. Sir Robert Hudson has rendered yeoman service during the war. He has attracted around him a band of financial administrators who have exercised control over nine million pounds of Red Cross money with wisdom and skill, by blending economy with full efficiency, repressing extravagance, but avoiding any crabbing, by niggardliness, of the supreme work for sick and wounded warriors. The work accomplished is declared to be of the best, and the key to its success, the guiding hand, the personal control of Sir Robert Hudson, who suffers no single branch or item of Red Cross activity to evade his personal knowledge, nor any detail to escape him. The methods of Sir Robert Hudson are clear and direct, resting on the widest of sympathies. He brooks no delays, and each day has to see its own work completely accomplished. His age and health place him in the full vigour and energy of his manhood. He has won for himself a multitude of friends and admirers, and has rendered services of supreme value to the nation and the Red Cross. As Chairman of the Finance Committee he is to be found at 83 Pall Mall, every day, directing the affairs of the greatest voluntary organisation for relief and succour, that the Empire has yet known. The country welcomed the King's decision to confer the Grand Cross of the Order of the British Empire upon Sir Robert Hudson for services, which can be appraised and appreciated by everybody throughout the nation.

### A NOTABLE MASONIC EVENT.

To hospital men who are also Freemasons, Friday, January 11, 1918, was a red-letter day, for on that date one who has been a member of the craft and a prominent hospital worker for half a century was installed Worshipful Master of a (or should we say the?) Hospital Lodge. Amid all the pomp and circumstance that Masonry permits itself during war-time, Sir Perceval Alleyn Nairne became Master of the Nosocomia Lodge, of which body he was a founder, and has since been a particularly honoured and beloved member. We do not betray any Masonic secrets, even if we were in possession of them, when we mention that Sir Perceval Nairne celebrated his jubilee in Freemasonry several years ago, when his portrait in oils, showing the subject in the regalia of a Past Grand Deacon of England, was presented to him, and when the recipient had the unusual, if not unique, honour of occupying the chair of his mother lodge fifty years after his initiation. With Sir Perceval's career as a hospital committee-man, deputy chairman, and chairman, at the Seamen's Hospital, Greenwich, our readers are familiar. A speaker at the lodge dinner last week said that Sir Perceval had been honoured by the King and by the Grand Master of Freemasons, but he possessed a distinction which came from neither of those powers—that of being a most loyal friend and a kindly and courtly gentleman. The thought was well expressed, and, moreover, it had the merit of being unreservedly true. The Nosocomia Lodge is fortunate, and should have a happy and prosperous year under its new presidency.

### DEATH OF THE LATE CHAIRMAN OF MOUNT VERNON HOSPITAL

By the death of Mr. Charles Johnston, at a comparatively early age, the Mount Vernon Hospital loses a warm and generous friend, whose association with the hospital dates back to the year 1890, when Mr. Johnston became a member of the committee of management, under the chairmanship of the late Mr. Benjamin Lyon. As might be expected in a business man, Mr. Johnston's voluntary service was not merely nominal. As a member of the finance committee his advice and assistance were always helpful and advantageous, and on the death of Mr. W. F. Malcolm in 1909 he succeeded to the chairmanship of the committee of management, a position he retained until his election to that of chairman of the hospital in 1911. That position he continued to occupy until December last, when, owing to a long and trying illness which rendered attendance at committee meetings impossible, Mr. Johnston retired, much to the regret of the committee and the medical staff of the hospital. His chairmanship was not uneventful. The passing of the National Health Insurance Acts and the uncertainty in regard to the future of chest hospitals rendered it necessary for the governing body to consider their position, and in the events which followed Mr. Johnston took a prominent part. The concentration of the work at Northwood interested him greatly, and the committee owe much to his foresight, which, with that of another prominent member of the committee, the late Mr. Charles D. Rudd, was instrumental in developing their present policy. He was largely concerned in the arrangements for the provision of the new wing at Northwood, which, when completed, will accommodate another 120 patients. It was his constant hope that he might live to see the completion of the building at the end of the war. His relations with the medical board were of the best, and by his death the committee of management and medical staff have lost an admirable colleague and chairman, and the staff a sincere friend.

### THE CASE AGAINST THE PENSIONS MINISTER.

The Ministry of Pensions lately challenged the criticisms of Sir Frederick Milner, whose indefatigable public spirit deserves the highest praise. The official reply, which amounted to saying that the situation was well in hand, should be contrasted with the definite statement of the deadlock in official procedure lately described by Sir Frederick in the newspapers. "It is utterly impossible," he says, "for the Pensions Ministry, hampered as they are by red-tape regulations, to deal with the daily increasing mass of cases that pass through their hands. They practically admit this themselves by stating that men who are dissatisfied must appeal to their War Pensions Committees; but what is the use of this if the Committees can only appeal back to the Pensions Ministry and wait for weeks and months before they can even get an answer to their letters, and then be asked to send copies of their original letters, which apparently have been mislaid, and no wonder?" A statement so damaging as this tears the official attitude to tatters. There can be no answer to Sir Frederick Milner so long as the present chaos continues. Indeed, the Pensions Minister has been placed in the humiliating position of sending out an official circular imploring Pensions Committees not to write about cases to him! We hope that he will be snowed under with letters until he either decentralises his responsibility or shows himself capable of doing the work.

### CAPITATION GRANTS FOR MILITARY OUT-PATIENTS.

The rates of payment to auxiliary hospitals in respect of out-patients billeted with or without subsistence are published in a recent Army Council instruction. Convalescent patients in auxiliary hospitals which receive a capitation grant from Army funds may be billeted at the maximum rate of 2s. 6d. per day for out-patients who continue to attend for medical treatment, and at 6d. per day for those patients billeted with subsistence who continue to attend hospital for treatment only. Auxiliary hospitals should not extend their present accommodation without instruction from Command Headquarters. It should be noticed that this instruction merely specifies the conditions for the payment of capitation grants to hospitals where such arrangements have been sanctioned. It does not give authority to billet soldiers undergoing hospital treatment.

### THE IMPERIAL WAR EXHIBITION.

The work of artists in the war is becoming recognised since their special gifts have been placed at the disposal of the wounded in a variety of unforeseen ways. Their work at the 3rd London General Hospital is also well known through the admirable gazette of that institution, and of late we have seen their employment by the different Governments even in their own work in connection with war memorials and paintings. In opening the Imperial War Exhibition at Burlington House in aid of the British Red Cross in France, Lord French went out of his way to pay a glowing tribute to the Artists' Rifles, who, he said, had rendered him special help in regard to the provision of officers and were one of the first Territorial regiments to arrive in France. The exhibition itself deserves a special article. Every military implement is represented. One photograph of naval guns in action was taken at night by their own flashes. The exhibition should do something to bring home to us in England the practical business of life at the Front, the pictorial aspects of which the artists in khaki have already done much to preserve.

### EXTENSION BY HUT.

The waiting-list in connection with the Victoria Hospital, Burnley, is growing so large that a modified scheme of extension is under consideration. The full scheme has been postponed till after the war, but meantime it is hoped that either by means of temporary buildings or in neighbouring buildings room may be found. The former solution is the more probable, and in view of the relative cheapness and convenience of these huts the main financial difficulty is that of maintenance. Mr. E. Halstead has made the position plain, and we believe that the public will support him. Twenty additional beds, and there are two hundred and seventy cases waiting, would cost £1,600 a year. On the other hand, as he justly remarked, "no hospital with a large waiting-list is performing its proper functions to the public." Since the building fund for the original scheme has already been secured for some time, the hospital can make an honest claim for having been prevented, in spite of itself, from meeting the present difficulty.

### GAZA HOSPITAL

Mr. H. G. Harding has written an account of the condition of Gaza Hospital after our recent bombardment, which supplements the statement that lately appeared in our columns. Writing to the *Church Family Newspaper*, he says that owing to the position of the hospital, which was next to a mosque used by the Turks as an ammunition store, direct hits on the latter damaged the hospital severely. The building, which was apparently in the direct line of fire, "suffered severely from our own shells." He adds that "it is in a ruinous condition, roofs off, gaping holes in the walls, the south wall of the operating theatre almost destroyed, and the west wall cracked from top to bottom. The floors are covered with piles of masonry, from which here and there fragments of broken bedsteads can be seen protruding. The doctor's house is so damaged that it threatens to collapse at any moment. Hence the whole place will have to be rebuilt." The hospital at Jaffa, Mr. Harding says, is untouched, though every shred of equipment has vanished. A sum of perhaps £7,500 is required to rebuild the Gaza institution. We cannot help saying that this sum would be much more readily raised were it not the custom of the Church Missionary Society to bury the doings of its hospitals from public knowledge. We have often done our best to publish such meagre information as is usually available.

### THE SALE OF SECRET REMEDIES IN FRANCE.

Though never enforced, the Code Napoléon provides penalties for the sale of remedies of unknown composition, and all who advertise or offer for sale such preparations are liable to imprisonment. But for many years the traffic in secret remedies has gone on unchecked, and their number is now estimated at over 40,000. For some weeks past the French Academy of Medicine have been considering their reply to a question proposed by the Ministry of the Interior on the subject of the control of the sale of these preparations. Naturally the question is not an easy one. Large sums of money and vested interests of all kinds are at stake, and with the object of maintaining the existing situation pressure is being brought to bear on both the medical and the lay press by the manufacturing firms. Two alternative suggestions have been put forward. The one, to authorise the sale of a secret remedy provided it has been examined by a special committee of the Academy and its formula deposited with the Academy; the other to prohibit the sale of any remedy whose exact formula is not published on the label. The latter view has attracted many adherents, and gains additional support from the fact that whilst the sale and export of secret remedies of French manufacture is tolerated, similar foreign preparations are seized by the Customs authorities. Remedies of standard and recognised worth would, it is maintained, lose nothing by the publication of their formulas, whilst under the same discipline the worthlessness of many widely advertised articles would become apparent.

### SALVATIONISTS IN FRANCE.

Amongst the many organisations, whether connected with the Established Church or with other bodies, which minister to the physical and spiritual welfare of our men across the water, few have such a record of unostentatious service ungrudgingly performed as that of which General Booth is the leader. The Salvation Army in pre-war days might not inaptly have been regarded as the spiritual counterpart of the "contemptible little army" that saved Europe at the Marne. Always in constant readiness for social service of every kind, leading lives of self-denial, not infrequently the victims of derision and misrepresentation, they yet proved their mettle against stern odds. It is not unnatural, therefore, that we should find them at the present time giving freely of their funds and *personnel* to relieve suffering and bring comfort to the British wounded. A further contingent of ambulance drivers has just been dedicated by General Booth. They are leaving shortly for France, and at the same time a cheque for the purchase and equipment of an ambulance unit has been presented to the Red Cross. General Booth, in his address, recalled Queen Alexandra's comment on the occasion of an inspection at Marlborough House of a previous unit. "Your idea is splendid. Not only are you sending over the right kind of cars; you are also sending over the right kind of men to drive them." No appreciation of the Salvation Army could express more tersely the peculiar value of their services.

### A LITERARY POINT.

Hospital secretaries and others must have long felt the need of a word that could be used to denote the period during which a patient is resident in hospital. There appears to be no word that quite covers the meaning desired, and the phrase officially employed by the King's Fund—" average number of days each patient was resident "—is apparently the best that can be done. The difficulty has been heroically tackled by the clerk to a London Pensions Committee, who recently wrote to the secretary of a hospital as follows:—" In confirmation of my telephonic communication of this morning, relating to the admittance of the above man into your hospital, I wish to say that my committee will be entirely responsible during the man's incarceration." One cannot regard the use of such a term, with its sinister associations, as happy, in connection with a discharged soldier; nor is its employment here strictly accurate, as the word used implies forcible detention. One shudders to think of the dark things that would be imagined of hospitals if their reports contained such phrases as " average period of *incarceration*." Perhaps the most convenient word is " stay," and it is easy to speak of the " average stay."

### INSTITUTION FIRE PRECAUTIONS.

Since the fatal fire at Crumpsall House, Manchester, most managers of such institutions have been reviewing the fire-prevention arrangements, and the fact that many have found it advisable to take extra precautions suggests that many of the arrangements have not been perfect. For example, the Birmingham Guardians communicated with the heads of the various institutions under their control and invited suggestions for improving the arrangements for fire prevention. As a result the Board has since given instructions for supplying fire extinguishers at each institution. At Marston Green and Erdington Homes the staff receive instruction once a month from a fire brigade official. After all, an actual fire alone provides a complete test of the adequacy of fire-prevention arrangements, but the Manchester calamity has caused all authorities to do anything that is humanly possible to prevent such disasters.

### GENEROUS ASSISTANCE TO MATERNITY AND CHILD-WELFARE SCHEMES.

Lambeth Borough Council have always taken a keen interest in maternity and child-welfare schemes, and some time ago decided to make grants out of the rates to voluntary agencies where Dr. J. Priestley, the medical officer of health, is satisfied that the work is carried on successfully. The Local Government Board have notified borough councils that they are prepared to make grants up to 50 per cent. of the expenditure on these organisations, and consequently the Lambeth Borough Council have set aside £400 for distribution. Already they have made the following grants: North Lambeth Babies' Care, 141 Kennington Road, £101 8s. 7d.; West Norwood Maternity Babies' Welfare Centre, £38 19s. 3d.; Brixton Infant Welfare Centre, Water Lane, £30 19s. 5d.; Moffatt Maternity and Child Welfare Centre, Esher Street, Upper Kennington Lane, £37 7s. 10d.; and the Brixton *Crèche*, 71 Effra Road, £20. Recently Dr. F. J. Waldo, Coroner for the City, laid particular emphasis on the great need there is for more *crèches* in London, as so many women are now compelled to go to work. It is probable that if these grants were more widely distributed there would be the desired increase in the number of *crèches* in the immediate future.

### ABERTILLERY HOSPITAL.

Last week a deputation representing the Abertillery Hospital Committee, and including the Right Hon. Tom Richards, M.P. for West Monmouth, visited the Ministry of Munitions in order to press home the urgent need for increased hospital accommodation in their town, with a view to inducing the Department to grant immediately the permits necessary to obtain materials to build a temporary hospital. Mr. Brace, M.P., introduced the deputation, which was received by Mr. Newton, head of the Materials Department of the Ministry, who made a sympathetic reply and promised an early decision in the matter.

### THE IMPORTANCE OF THE CRECHE.

Dudley Corporation seems fully alive to the needs of the times. Not long ago it decided to have Corporation midwives, and three of these are in practice. It is now found that mothers attending the Maternity and Child Welfare Centre are often unable to get enough milk for their babies, and so the medical officer is suggesting that a milk depôt should be established. He also finds that there is need for a *crèche* for infants whose mothers have to go to work. There is an urgent demand for such accommodation among the working people. In many instances considerable sums are paid weekly by parents for the care of their children, who are to some extent neglected. The cost of a municipal *crèche* would not be a great burden on the rates, as 75 per cent. of the cost is repayable by the Local Government Board.

### THIS WEEK'S DRUG MARKET

A fair amount of business is being transacted, and the tendency of prices is still in an upward direction. The shortage of bromide of potassium is more pronounced, and higher prices are asked. Acetanilide is again dearer, and supplies are getting scarcer. The upward movement in the price of hexamine continues. Business has been done in phenacetin at what is nowadays a very low price, but as recent buying has reduced stocks considerably, the price tendency is now upwards. Balsam Tolu is dearer. A fair quantity of quinine has changed hands, and, in view of the depleted stocks, it is rather suspicious that the price does not advance more substantially. A census of the stocks of some hundred or so commonly prescribed drugs is being taken, and it is not unexpected that the Government control of drugs will become tighter.

